# Performance of the low-cost phenotypic thin-layer agar MDR/XDR-TB Colour Test (first generation, 1G, Color Plate Test) for identifying drug-resistant Mycobacterium tuberculosis isolates in a resource-limited setting

**DOI:** 10.21203/rs.3.rs-6697928/v1

**Published:** 2025-06-27

**Authors:** Binyam Mebrat, Juan I. Garcia, Yimtubezenash Woldeamanuel, Kelemework Adane, Amberlee Hicks, Melaku Tilahun, Sebsib Neway, Lelisa Oluma, Abay Atnafu, Jonathan Gelfond, Carlton A. Evans, Jordi B. Torrelles, Shu-Hua Wang, Liya Wassie

**Affiliations:** Armauer Hansen Research Institute (AHRI); Texas Biomedical Research Institute; Addis Ababa University; Addis Ababa University; Texas Biomedical Research Institute; Armauer Hansen Research Institute (AHRI); Armauer Hansen Research Institute (AHRI); Armauer Hansen Research Institute (AHRI); Armauer Hansen Research Institute (AHRI); UT Health San Antonio; IFHAD: Innovation for Health And Development, Imperial College London; Texas Biomedical Research Institute; Texas Biomedical Research Institute; Armauer Hansen Research Institute (AHRI)

**Keywords:** Mycobacterium tuberculosis, drug-resistant TB, diagnosis, drug susceptibility test, thin-layer agar, MDR/XDR-TB Colour Test, First generation (1G) Color Plate Test

## Abstract

**Background:**

The accessible, easy to use and timely, diagnosis of tuberculosis (TB) drug-susceptibility, including multi-drug resistant (MDR-) TB and extensively-drug resistant (XDR-)TB is often challenging, particularly in resource-constrained settings. We therefore evaluated the phenotypic thin-layer agar based MDR/XDR-TB Colour Test, which is also referred to as the “First Generation (1G) Color Plate Test (TB-CX)” performance for detecting resistance of *Mycobacterium tuberculosis* (*Mtb*) isolates to selected anti-TB drugs versus other tests routinely used in our setting.

**Methods:**

A cross-sectional study was conducted on *Mtb* clinical isolates stored at the Armauer Hansen Research Institute TB laboratory in Addis Ababa, Ethiopia. Drug-susceptibility testing was performed on 78 *Mtb* isolates for isoniazid, rifampicin, and moxifloxacin using the Colour Test and the Indirect Proportional Method (IPM) “in house” assay. Isoniazid and rifampicin were also evaluated by the Mycobacterial Growth Indicator Tube (MGIT) commercially available assay. Test accuracy was calculated as % agreement with 95% confidence intervals (95%CI).

**Results:**

The median (range) times in days determining *Mtb* resistance or susceptibility for the Colour Test, IPM and MGIT assays were of 9 (5–18), 15 (13–18) and 18 (14–21) days, respectively. The Colour Test provided results significantly (p < 0.001) more rapidly than the IPM or MGIT assays. Colour Test accuracy compared to MGIT DST for detecting isoniazid and rifampicin resistance and MDR-TB was 88% (95%CI = 81–96), 92% (95%CI = 86–98), and 94% (95%CI = 88–99), respectively. Colour Test accuracy compared to IPM to detect isoniazid, rifampicin resistance and MDR-TB was 92% (95%CI = 86–98), 81% (95%CI = 72–90), and 90% (95%CI = 83–96). IPM test accuracy compared to MGIT DST for detecting isoniazid and rifampicin resistance and MDR-TB was 91% (95%CI = 85–97), 83% (95%CI = 75–92), and 85% (95%CI = 77–93), respectively. Moxifloxacin drug-susceptibility testing could not be assessed because only two isolates showed evidence of resistance.

**Conclusion:**

The accuracy of *Mtb* drug-susceptibility testing was similar comparing: Colour Test versus IPM, Colour Test versus MGIT; and comparing IPM versus MGIT. The Colour Test was easy to use and determined drug-susceptibility significantly more rapidly than the IPM and MGIT assays. Thus, implementing the Colour Test in clinical settings could make drug-susceptibility testing more accessible and rapid in high TB burden, and resource-constrained settings, including in Ethiopia.

## INTRODUCTION

Tuberculosis (TB) is caused by *Mycobacterium tuberculosis* (*Mtb*) complex (1). The World Health Organization (WHO) has estimated that in 2023 there were 10.8 million cases, resulting in 1.25 million deaths, including 161,000 deaths in individuals living with HIV (2). With the decline of COVID-19, TB is once again the world’s most frequent cause of death from a single infectious agent and caused almost twice as many deaths as HIV/AIDS(3).

Difficulties in TB diagnosis are important barriers to global attempts to end the TB pandemic (4). Due partly to limitations in the timely diagnosis of drug-resistant (DR)-TB, and a shortage of phenotypic and genotypic tools in resource-limited areas, multi-drug-resistant (MDR)-TB is increasing globally (5). In recent years, the development and evaluation of rapid and low-cost phenotypic culture methods have been considered to be priorities to fulfil ‘The End TB Strategy’ milestones (6). These include liquid media-based culture, microscopic observation drug susceptibility assay (MODS), the nitrate reductase assay, and the colorimetric redox-indicator assays (7–9).

Although genotypic methods of TB diagnosis are effective, rapid, and detect resistant clinical isolates (10), they are often costly and some require skilled personnel and also sophisticated equipment and maintenance (11). A low-cost, phenotypic method that has been developed is the thin layer agar (TLA) based MDR/XDR-TB Colour Test (abbreviated to “TB-CX”), which is also referred to as the “First Generation (1G) Color Plate Test (TB-CX)” (12, 13). The TB-CX is designed for use in resource-constrained settings to provide timely, relatively bio secure and accurate results to complement or replace conventional culture methods in the field, such as the Mycobacteria growth indicator tube (MGIT) DST assay, and Löwenstein-Jensen (LJ) culture DST. This has the potential to facilitate early TB treatment of patients with MDR-TB (13), either by direct testing of specimens collected from patients (most frequently sputum), or by indirect testing of *Mtb* isolates that have already been cultured from patient specimens.

The current study aimed to evaluate the performance of the TB-CX to detect drug resistance in *Mtb* by indirect testing of isolates compared to two existing DST methods: the indirect proportion method in 7H10 agar (IPM assay) and the MGIT assay.

## METHODS

### Study setting

A cross-sectional study was conducted on stored *Mtb* clinical isolates, archived at the Armauer Hansen Research Institute (AHRI) TB laboratory, in Ethiopia, from 2020 to 2022. A purposive sampling method that was focused on the quality of samples by evaluating storage condition, the absence of leakage and availability of linked clinical data was used to select 105 *Mtb* stored isolates from the TB laboratory repository.

Demographic and clinical data, including age, sex, and data on previous history of anti-TB treatment were collected using a standard data collection form. Ethical approval was obtained from the Research Ethics Committee of the Department of Microbiology, Immunology and Parasitology, College of Health Sciences, Addis Ababa University, and the AHRI/All Africa Leprosy and Tuberculosis Rehabilitation and Training Center (ALERT) Ethics Review Committee (Protocol number PO-07/23). All data were de-identified, and only coded samples and their linked clinical data were used in the study.

### Laboratory preparation ofMtbbacterial suspensions for DST

*Mtb* clinical isolates were stored in cryopreservation media (*i.e*., 7H9 broth supplemented with a final concentration of 25% glycerol), to optimise preservation. Additionally, the quality of storage conditions was rigorously evaluated and used as a critical criterion in the selection process. Selected *Mtb* clinical isolates (n = 105) were sub-cultured on LJ media. Those that grew on LJ culture media (n = 78) were then considered for further analyses.

Colonies were identified and suspended in 5 drops (~ 200 μl) of sterile distilled water. The tubes were shaken by hand for 1 min and suspensions were allowed to settle for 15 min. A *Mtb* suspension with an optical density (assessed by eye as cloudiness) of McFarland grade 0.5 was prepared for inoculation of MGIT DST and McFarland 1 was prepared and diluted accordingly for the inoculation of the TB-CX and IPM assays.

### Laboratory assessment of DST

Three laboratory assays were used for laboratory assessment of DST: the TB-CX and IPM assay for isoniazid, rifampicin and moxifloxacin DST, and MGIT DST for isoniazid and rifampicin only.

### The TB-CX

**Figure 1:**
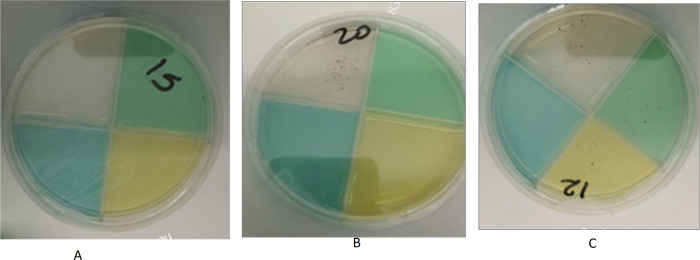
**A:** The TB-CX with the drug susceptible detection quadrant (clear), and isoniazid-resistant (green), rifampicin-resistant (yellow), and moxifloxacin-resistant (blue) quadrants. 1B: Susceptible: *Mtb*growth on drug-free quadrant (red colonies, top, clear) and susceptible to all three drugs tested. 1C: MDR-TB: Concurrent *Mtb* growth on drug-free quadrant (top, clear), green isoniazid- containing quadrant, and yellow rifampicin-containing quadrant indicating resistance to isoniazid and rifampicin and susceptibility to moxifloxacin.

This test was manufactured at Texas Biomedical Research Institute, in San Antonio, Texas and shipped to Ethiopia within 2 weeks of manufacture at room temperature. All TB-CXs were shipped together in two batches to Ethiopia and this shipping took approximately 5 days and the TB-CX was shipped as commercial airline ‘hold’ baggage in standard suitcases, so may have been subjected to freezing and or high temperatures in transit. Upon arrival, plates were stored at 2 to 8°C until ready to use (9, 12–15). Including transit time, the interval between manufacture of the TB-CX and their inoculation was up to 35 days. The TB-CX is based on a thin layer of 7H11 agar media containing oleic acid, albumin, dextrose, catalase (OADC), glycerol, and selective agents to inhibit other bacterial and fungal growth and a colorimetric indicator of microbial growth (12). The TB-CX contains four quadrants: a clear ‘control’ quadrant not containing any anti-TB drug, and the 3 other quadrants containing isoniazid, rifampicin, and moxifloxacin, following the critical concentrations (CC) recommended by the WHO for 7H11 of 0.2 μg/ml for isoniazid, 1.0 μg/ml for rifampicin, and 0.5 μg/ml for moxifloxacin. To distinguish drug-containing quadrants a food colorant was added (green food colorant for isoniazid quadrant, yellow for rifampicin’s quadrant and blue for moxifloxacin quadrant). (Fig. 1A). *Mtb* growth on the TB-CX was identified by visualizing red colonies with naked eye or using a 40X magnifier (13, 14). The TB-CXs were inoculated with two drops (~ 80 μl) 1:10 and 1:100 of dilutions of McFarland 1 suspensions in each quadrant for each isolate (15). Each TB-CX was inspected for red colonies until at least 11 colonies were seen in the control quadrant or until 42 days after inoculation (9, 12, 14, 15). To detect **drug susceptibility** a minimum of eleven red colonies needed to be observed in the control quadrant and no red colonies in the drug containing quadrant (Fig. 1B). To detect **drug resistance** a minimum of eleven red colonies need to be observed in the control quadrant and at least growth of one red colony in the drug containing quadrant. The TB-CX has similarities with the agar proportion method with the 1% critical threshold (15). By definition, **mono-drug resistance** for isoniazid, rifampicin, or moxifloxacin were defined when growth was observed in the control quadrant and concurrently with growth in the drug-containing quadrants. **MDR-TB** was defined when concurrent growth occurred in the control, isoniazid, and rifampicin quadrants. **Pre-XDR TB** was defined when concurrent growth occurred in the control, isoniazid, rifampicin, and moxifloxacin quadrants (Fig. 1C). However, resistance/susceptibility was considered uninterpretable if the number of colonies in the drug containing quadrant was less than 1% of the colony count in the detection quadrant. A DST result was reported as **uninterpretable** if less than 11 colonies grew in the control quadrant until 42 days of incubation, regardless of growth in the drug-containing quadrant. An isolate was considered to be **negative** for *Mtb* if there was no growth on any of the quadrants for 42 days. Both batches of TB-CX underwent quality control in Texas and Ethiopia that involved analysis using H37Rv pan susceptible strain and a strain with known drug-resistance to isoniazid, rifampicin and moxifloxacin.

### The 24-well indirect proportion method (IPM)

**Figure 2:**
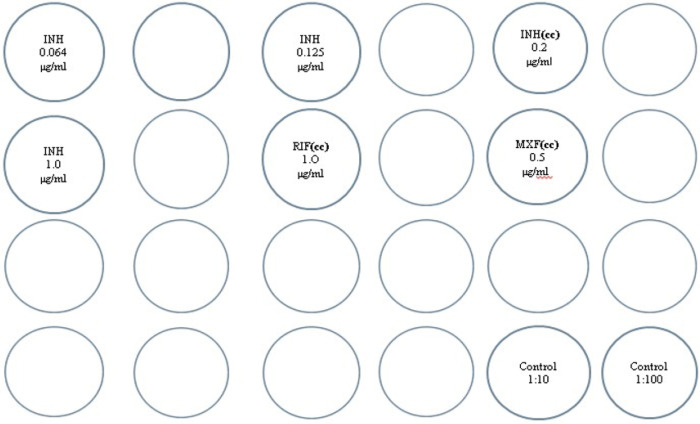
Schematic arrangement of the 24 well plate for the IPM test. Note CC indicates Critical Concentration.

A modified IPM assay adapted for 24-well agar plates containing 7H10 medium was used (16). Growth was evaluated according to the proportion method by comparing the 1:100 diluted controls to the drug containing wells as illustrated in Fig. 2. The following WHO CC for the IPM assay drugs were used to differentiate susceptible from resistant isolates: 0.2 μg/ml for isoniazid, 1.0 μg/ml for rifampicin, and 0.5 μg/ml for moxifloxacin (i.e., the same concentrations as were used in the TB-CX (16, 17). Plates were placed in a rack, covered with adhesive tape on the plates for safety reasons and incubated at 37°C for about 21 days. A strain was reported susceptible if there was more growth in the 1:100 diluted control than in the drug-containing well with the CC of the drug; and resistant, if there was more growth in the drug-containing well containing the CC than in the 1:100 diluted control. The test was repeated if there was equal growth (16).

### MGIT DST

MGIT SIRE (streptomycin, isoniazid, rifampicin and ethambutol) kits were processed using BACTEC MGIT 960 mycobacterial detection instrument (BACTEC MGIT 960, BD Diagnostics, Franklin Lakes, NJ) according to manufacturer′s guidelines, and using isoniazid and rifampicin CC of 0.1 μg/ml and 1 μg/ml, respectively (i.e., a lower isoniazid and equal rifampicin concentration to those used in the TB-CX and IPM) (13, 14, 17). *Mtb* isolates were sub-cultured and incubated at 37°C in the MGIT equipment, until the device indicated a positive tube. DST was performed using one 7-ml MGIT tube as a control and another tube for each of isoniazid and rifampicin. When 1% or more of the test population grew in the presence of the CC of the drug, an isolate was defined as resistant (16).

### Analysis

Time-to-culture growth for DST interpretation was calculated in days from the date of inoculation to the date of interpretable/valid results. Data were entered into Excel spread sheets, cleaned, and imported to statistical package for social sciences (SPSS, version 27) for statistical analyses. Data that had a skewed distribution such as time to test positivity were summarized by their median and inter-quartile ranges (IQR) and compared. Data that had an approximately normal distribution were summarised by their mean and standard deviation (SD) or 95% confidence intervals (95%CI). Proportions were stated as percentages with their 95%Cl. All 95%CI were calculated using the Wald formula. The diagnostic accuracy of the TB-CX was evaluated by determining its accuracy, sensitivity, specificity, and positive and negative predictive values (PPV, NPV), using the IPM assay and MGIT DST as reference assays for comparison. Accuracy was defined as the proportion of all results that were correct (i.e., the sum of true positive and true negative results divided among all results and expressed as a percentage) (18). Test agreements between the tests were analysed using κ-values. The two-sample Z-test of proportions was used to compare the proportions and their 95%CI for the different diagnostic tests. The McNemar chi^2^ test was also used to assess the statistical significance of specific paired comparisons.

## RESULTS

The characteristics of the study population are shown in Table 1. 78 isolates were derived from patients who were 55% male, with a mean age of 30 years (SD = 14), and 52 (67%) arose from sputum samples of pulmonary TB patients whereas 26 (33%) were isolated from lymph node aspirates from patients with extra-pulmonary TB. The majority of the samples analysed, 62/78 (79%), were collected from newly diagnosed TB patients; of the remaining 6/78 (7.7%) had treatment failure, 5.1% (4/78) had a prior history of TB treatment with anti-TB medications, 3/78 (3.8%) were relapse and 3/78 (3.8%) had unknown TB treatment outcomes results. 29/78 (37%) were unknown where as 10% (5/49) of the *Mtb* isolates were collected from people living with HIV (PLWH),other.

### Performance evaluation of the TB-CX vs. IPM

The TB-CX sensitivity compared to IPM to detect isoniazid, rifampicin, and moxifloxacin resistance alone or isoniazid combined with rifampicin (MDR-TB) was 97%, 82%, 100%, and 100%, respectively, and its specificity was 89%, 80%, 99%, and 86%, respectively. The TB-CX diagnostic accuracy for isoniazid, rifampicin, and moxifloxacin resistance detection, and MDR-TB detection was 92%, 81%, 97%, and 90%, respectively. Agreement between the TB-CX and IPM had a kappa value of 0.84 (Table 2).

### Performance evaluation of the TB-CX vs. MGIT

The agreement between the TB-CX and MGIT DST to detect isoniazid, rifampicin, and MDR-TB were 88%, 92% and 94%, respectively (Table 3). For the detection of isoniazid, rifampicin resistance, and MDR-TB, the performance of the TB-CX compared to the reference MGIT, yielded a sensitivity of 91%, 93%, and 90%, and specificity of 87%, 92%, and 96%, respectively.

### Performance evaluation of the IPM vs. MGIT

When compared to the MGIT DST, the sensitivity and specificity of IPM for detecting isoniazid resistance was 88% and 94%, respectively; and for detecting rifampicin resistance was 73% and 90%, respectively (Table 4). In addition, the sensitivity and specificity of IPM for detecting MDR-TB was 72% and 96%, respectively. The agreement between IPM and MGIT was 91%, 83% and 85%, respectively.

### Time to Mtb Culture Growth Detection for DST

**Figure 3:**
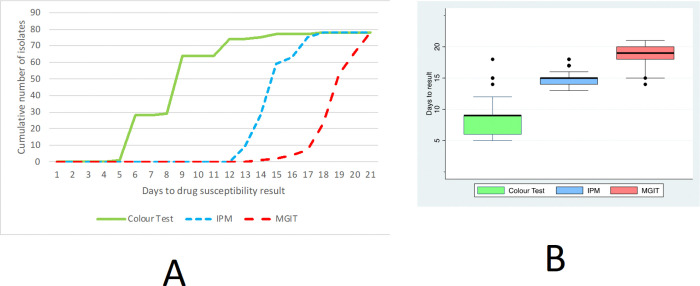
A. The turnaround time days from sample processing until the results of the three drug-susceptibility tests used in this study. Note: IPM indicates the Indirect Proportional Method assay; and MGIT indicates the Mycobacterial Growth Indicator Tube (MGIT) assay. 3B. A box plot summarising the turnaround time days from sample processing until the results of the three drug-susceptibility tests used in this study. Note: In this standard box plot, the thick lines indicate medians, boxes indicate interquartile ranges, “whiskers” indicate upper/lower adjacent values equal to the 75^th^/25th percentile plus/minus 1.5 times the interquartile range, and dots indicate outliers; IPM indicates the Indirect Proportional Method assay; and MGIT indicates the Mycobacterial Growth Indicator Tube (MGIT) assay.

The turnaround time in days for the TB-CX ranged 5–18 (mean 8.6, median 9, IQR = 6–9, SD = 2.6); for IPM ranged 13–19 (mean 15, median 15, IQR = 15, SD = 0.90); and for MGIT ranged 14–21 (mean 19, median 19, IQR = 18–20, SD = 1.4) (Fig. 3A&3B). The turnaround time for the TB-CX was significantly (p < 0.001) faster than the IPM and then the MGIT test.

### Comparative analysis of DST methods for isoniazid, rifampicin and MDR-TB

**Figure 4:**
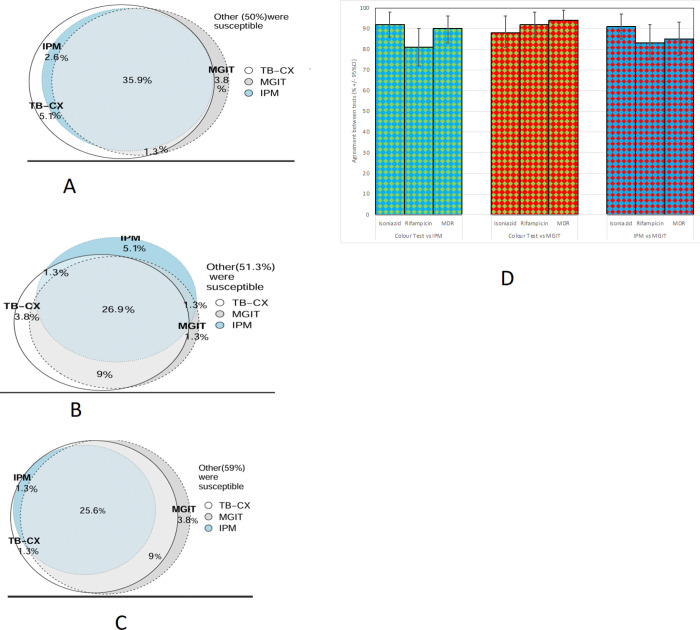
A. Venn diagram for isoniazid resistance detection 4B. Venn diagram for rifampicin resistance detection 4C. Venn diagrm for MDR-TB detection by TB-CX, MGIT, and IPM 4D. The accuracy (percentage agreement) between the results of the three drug-susceptibility tests used in this research. Note: CI indicates confidence interval; MDR indicates multi-drug resistance; IPM indicates the Indirect Proportional Method assay; and MGIT indicates the Mycobacterial Growth Indicator Tube (MGIT) assay.

Among the 78 isolates tested, 50% (39/78) were classified as susceptible by all three DST methods (TB-CX, IPM, and MGIT). The TB-CX identified 5.1% (4/78) of isolates as isoniazid-resistant, despite being susceptible by both IPM and MGIT, with an overall agreement of 86% (67/78) in detecting isoniazid resistance (Fig. 4A). For rifampicin resistance, 47% (37/78) of the isolates were susceptible across all three methods, while 3.8% (3/78) were identified as rifampicin-resistant exclusively by the TB-CX method, resulting in an overall agreement of 78% (61/78) among the three methods (Figure B). Regarding MDR-TB, one isolate (1.3%, 1/78) was classified as MDR-TB solely by the TB-CX method, with an overall agreement of 85% (66/78) among the three DST methods (Fig. 4C&Figure 4D). Additionally, for moxifloxacin susceptibility, 97% of the isolates were susceptible in both the IPM and TB-CX methods, while 1.3% (1/78) were resistant only in the TB-CX method, and another 1.3% (1/78) were resistant in both the TB-CX and MGIT methods. Notably, moxifloxacin was not tested using the MGIT method.

## DISCUSSION

This study evaluated the performance of the TB-CX as an alternative testing method for the detection of *Mtb* resistance to isoniazid, rifampicin, and moxifloxacin in clinical isolates. When compared to IPM and MGIT DST, the TB-CX showed good performance (sensitivity, specificity and diagnostic accuracy) in detecting resistance to isoniazid, rifampicin, moxifloxacin and MDR-TB. The TB-CX agreed with IPM and MGIT with similar frequency to the IPM and MGIT agreed with each other.

Our results indicate that the time to determine *Mtb* resistance/susceptibility was significantly shorter using the TB-CX compared to IPM and MGIT, an important advantage of the TB-CX in early detection of *Mtb* drug resistance. The turnaround time of the TB-CX in this study was slightly shorter than previously reported (9, 19), which may be due to the higher bacterial load in the 1 McFarland bacterial suspension compared to that in most sputum samples. In this study, stored *Mtb* isolates were sub-cultured in LJ tubes, and subsequently used for both the TB-CX and IPM test.

Specifically, compared to MGIT DST, a gold standard for phenotypic DST, the TB-CX exhibited good sensitivity (91%) for detecting isoniazid resistance, but was relatively lower than the reported 98% in a study conducted in Estonia using 201 archived *Mtb* clinical isolates (12). Additionally, the TB-CX showed strong sensitivity (93%) and specificity (83%) for detecting rifampicin resistance, which was comparable with the 98% sensitivity observed in similar studies comparing the TB-CX with MGIT DST (9, 12). The TB-CX had 100% sensitivity for the detection of MDR-TB, comparable to other studies at 95–99% as well as 86% specificity; although this was slightly lower than reported in other similar studies (9, 12). When compared to IPM assay, it also showed excellent sensitivity (97%) and specificity (90%) for detecting MDR-TB plus moxifloxacin resistant isolates, with a 93% agreement rate.

Interestingly, using the same samples, the sensitivity of the TB-CX to detect rifampicin resistance (93%) was greater than the sensitivity of the IPM test to detect rifampicin resistance (73%) using MGIT as a reference standard. The rifampicin CC of 1 μg/ml used in the IPM test was higher than the 0.5 μg/ml recommended in the latest WHO technical report on CC rifampicin for 7H10 media; however, only limited data are available to determine if the rifampicin CC for 7H11 could be lowered, as reported for 7H10 (20). The increased CC threshold in the CC for the IPM test might explain the decreased sensitivity of the IPM assay to detect rifampicin resistance compared to the TB-CX, as we could expect an increase in false susceptible results (truly resistant strains misclassified as susceptible) and thus, lowering IPM sensitivity.

When compared to the IPM assay, the TB-CX demonstrated a sensitivity of 97% for detecting isoniazid-resistant *Mtb* isolates. Our study has shown a higher sensitivity than that reported in North East Ethiopia(9), where the sensitivity was only 59%. This could be explained by the fact that our study used archived clinical isolates with standardized inoculum suspensions that may increase the sensitivity of TB-CX when compared to decontaminated sputum samples. Comparable to other studies (9, 12, 21), the TB-CX showed a sensitivity of 82% and a specificity of 80% for rifampicin resistance detection when compared with MGIT DST.

Although no other studies evaluated moxifloxacin resistance, the TB-CX had a sensitivity of 100% and a specificity of 99% in detecting moxifloxacin resistance, which is comparable to a study that evaluated ciprofloxacin (9). These results show excellent agreement between 7H10/7H11 media as previously reported (22), although studies with larger numbers of quinolone resistant *Mtb* are needed for full assessment.

While TB culture is often prone to contamination, and hence resulting in misdiagnosis or missed diagnosis (14), a method that minimizes contaminations is ideal. The contamination rate for the reference IPM method was compared to the TB-CX. None of the isolates tested by TB-CX showed contaminations, but two of these isolates (2.4%) showed contamination with the IPM method. Similar to the current study, no contamination was reported with other TB-CX studies published previously (9, 12, 14). 7H11 agar has been proposed as an improvement of 7H10 agar (23, 24). The TB-CX has additional reagents to minimize contaminations, such as carbendazim, a fungicide, and Selectatab (11), which is a selective supplement used to suppress the growth of common sputum contaminants.

Despite the potential limitations of the study in using archived *Mtb* isolates and hence the lack of sensitivity results using fresh specimens (e.g. sputum or lymph node biopsy), our study showed that the TB-CX was quick and simple to use for identifying drug-susceptible, and drug-resistant (including MDR-TB), using stored clinical isolates with good sensitivity and specificity, with shorter time to positive results when compared to IPM and MGIT DST. Because the TB-CX is inexpensive (US$1.2), easy to use, and does not require highly trained personnel, it could be a reliable and economically viable option for DST in resource-limited settings. Diagnosing DR-TB with WHO-approved phenotypic and genotypic methods is often challenging in resource-limited settings, like Ethiopia, since some tests require expensive equipment like MGIT, GeneXpert, and/or sequencing equipment, which also require training, calibration, maintenance, and expensive supplies.

Future studies may evaluate the application of targeted next generation sequencing of DR-TB strains, to also identify *rpoB* mutations. Conventional phenotypic culture such as LJ and agar-based DST methods also have drawbacks as these take a longer time to deliver test results, which impact patient diagnosis, treatment, and follow-up (13). Future studies may consider implementing the TB-CX for DST of first and second-line anti-TB drugs other than moxifloxacin, using a larger sample size and a variety of other specimens, such as decontaminated sputum samples, stools (for paediatric TB diagnosis and DST), cerebrospinal fluid, and lymph node biopsies. If the TB-CX can improve accuracy in 1st or 2nd line drug resistance detection and turnaround time compared to MGIT SIRE, it could be a major advancement to simplify and decentralize DST capacities in high TB burden, resource constrained settings.

## Figures and Tables

**Table 1:** Characteristics of the study population.

Variable	Number	Measure	Data
Female sex (versus male)	43/77	%	56
		95%CI	45–67
Age (years)	77	Median	27
		IQR	23–34
		Range	0.5–70
HIV seropositive (versus seronegative)	5/49	%	10
		95%CI	1.7–19
Retreatment TB (versus new cases)	13/75	%	17
		95%CI	7.7–26
Fine needle aspirate sample (versus sputum)	26/78	%	33%
		95%CI	23–44

Note: as indicated by the “Number” column, some data were missing for some of the 78 samples; CI indicates confidence interval; IQR indicates inter-quartile range; and HIV indicates human immunodeficiency virus.

**Table 2:** Performance of the TB-CX *vs*. the reference standard IPM drug susceptibility test

TB-CX	IPM		Sensitivity% (95% CI)	Specificity% (95% C)	PPV% (95%C)	NPV% (95%CI)	Kappa value	Accuracy (95%CI)
Isoniazid	R	S						
R	30	5	97 (87–100)	89 (79–96)	86 (72–95)	98 (90–100)	0.80	92 (86–98)
S	1	42						
Rifampicin	R	S						
R	22	10	82 (64–93)	80 (68–90)	69 (52–83)	89 (78–96)	0.60	81 (72–90)
S	5	41						
MDR	R	S						
R	21	8	100	86 (75–93)	72 (55–86)	100	0.87	90 (83–96)
S	0	49						

**Note:** TB-CX = Tuberculosis colour test test; IPM = Indirect Proportional Method; PPV = Positive Predictive Value; NPV = Negative Predictive Value; R = resistant; S = susceptible; MDR = Multi Drug Resistance

**Table 3:** Performance of the TB-CX *vs*. the reference standard MGIT drug susceptibility test

TB-CX	MGIT		Sensitivity (95%CI)	Specificity (95%CI)	PPV (95%CI)	NPV (95%CI)	Kappa value	Accuracy (95%CI)
Isoniazid	R	S						
R	29	6	91 (76–98)	87 (75–95)	83 (68–93)	93 (83–98)	0.70	88 (81–96)
S	3	40						
Rifampicin	R	S						
R	28	4	93 (81–99)	92 (82–97)	88 (73–96)	96 (87–99)	0.80	92 (86–98)
S	2	44						
MDR	R	S						
R	27	2	90 (76–97)	96 (88–99)	93 (80–99)	94 (85–98)	0.87	94 (88–99)
S	3	46						

**Note:** MGIT = mycobacteria growth indicator tube; TB-CX = Tuberculosis colour plate test; MDR: multi-drug resistant, IPM = Indirect Proportional Method;PPV = Positive Predictive Value; NPV = Negative Predictive Value; R = resistant; S = susceptible

**Table 4:** Performance of IPM vs. MGIT drug susceptibility tests

IPM		MGIT		Sensitivity (95% CI)	specificity (95% CI)	PPV (95%CI)	NPV (95%CI)	kappa value	Accuracy (95%CI)
Isoniazid	R	R	S	88 (73–96)	94 (84–98)	90 (77–98)	92 (81–97)	0.80	91 (85–97)
		28	3						
	S	4	43						
Rifampicin		R	S						
	R	22	5	73 (56–87)	90 (79–96)	81 (56–81)	84 (77–93)	0.64	83 (75–92)
	S	8	43						
MDR	R	20	2	72 (56–85)	96 (88–99)	93 (80–99)	82 (70–91)	0.70	85 (77–93)
	S	10	46						

**Note:** IPM = indirect proportion method; MGIT = Mycobacteria Growth Indicator Tube; DST = Drug Susceptibility Test; PPV = positive predictive value; NPV = negative predictive value; R = resistant; S = susceptible; FNA = Fine Needle Aspiration.

## Data Availability

All relevant data are available at https://figshare.com/s/21153360d12e74afb786
